# Beneficial Effects of Different Types of Exercise on Diabetic Cardiomyopathy

**DOI:** 10.3390/biom15091223

**Published:** 2025-08-25

**Authors:** Xiaotong Ma, Haoyang Gao, Ze Wang, Danlin Zhu, Wei Dai, Mingyu Wu, Yifan Guo, Linlin Zhao, Weihua Xiao

**Affiliations:** 1 Shanghai Key Lab of Human Performance, Shanghai University of Sport, Shanghai 200438, China; 2321518007@sus.edu.cn (X.M.); 2411516001@sus.edu.cn (H.G.); 2221518047@sus.edu.cn (Z.W.); zdl981030@gmail.com (D.Z.); 2321518018@sus.edu.cn (W.D.); 2321517005@sus.edu.cn (M.W.); 2 School of Elderly Care Services and Management, Nanjing University of Chinese Medicine, Nanjing 210023, China; yifan_guo@njucm.edu.cn; 3 School of Physical Education, Shanghai Normal University, Shanghai 200234, China

**Keywords:** exercise intervention, type 2 diabetes mellitus, diabetic cardiomyopathy, inflammation, fibrosis, PANoptosis, cGAS-STING pathway

## Abstract

Diabetic cardiomyopathy (DCM) is a serious complication of type 2 diabetes mellitus (T2DM), characterized by cardiac dysfunction, inflammation, and fibrosis. In this study, a T2DM mouse model was established by administering a high-fat diet (60% fat) in combination with streptozotocin injection in male C57BL/6J mice. The mice subsequently underwent an eight-week exercise intervention consisting of swimming training, resistance training, or high-intensity interval training (HIIT). The results showed that all three forms of exercise improved cardiac function and attenuated myocardial hypertrophy in DCM mice. Exercise training further downregulated the expression of pro-inflammatory cytokines, including interleukin-6, tumor necrosis factor-α, nuclear factor κB, and monocyte chemoattractant protein-1, and mitigated myocardial fibrosis by suppressing fibronectin, α-SMA, collagen type I alpha 1 chain, collagen type III alpha 1 chain, and the TGF-β1/Smad signaling pathway. Moreover, exercise inhibited the expression of PANoptosis-related genes and proteins in cardiomyocytes of DCM mice. Notably, HIIT produced the most pronounced improvements across these pathological markers. In addition, all three exercise modalities effectively suppressed the aberrant activation of the cGAS–STING signaling pathway in the myocardium. In conclusion, exercise training exerts beneficial effects against DCM by improving cardiac function and reducing inflammation, PANoptosis, and fibrosis, and HIIT emerged as the most effective strategy.

## 1. Introduction

Type 2 diabetes mellitus (T2DM) is a chronic metabolic disorder characterized by persistent hyperglycemia resulting from inadequate insulin secretion or insulin resistance and is influenced by genetic and environmental factors [1,2]. Diabetic cardiomyopathy (DCM), a frequent and serious complication of diabetes, is defined as structural and functional abnormalities of the myocardium that occur independently of coronary artery disease, hypertension, or obesity [3].

A cross-sectional clinical study reported that 25.8% of patients with T2DM exhibited left ventricular dysfunction [4]. Similarly, animal studies have demonstrated that mice with T2DM induced by a high-fat diet (HFD) combined with streptozotocin (STZ) develop cardiac dysfunction and myocardial hypertrophy [5]. Furthermore, both type 1 diabetes mellitus and T2DM mouse models display myocardial inflammatory cell infiltration and increased cytoplasmic vacuolization [6]. DCM is closely associated with inflammatory cell activity [7]. Pro-inflammatory cytokines, including interleukin-6 (IL-6), tumor necrosis factor-α (TNF-α), interleukin-18 (IL-18), and the chemokine monocyte chemoattractant protein-1 (MCP1), have been implicated in its pathogenesis [8,9,10]. Notably, the silencing of the inflammatory mediator nuclear factor κB (NF-κB) has been shown to attenuate DCM by suppressing ferroptosis [11]. Myocardial fibrosis is a well-recognized pathological feature of DCM [12]. Clinical evidence has revealed elevated expression levels of collagen type I alpha 1 chain (Col1a1) and collagen type III alpha 1 chain (Col3a1) in patients with DCM, further indicating the presence of fibrosis [13]. Transforming growth factor beta (TGF-β), a classical profibrotic factor [14], plays a central role in this process, and numerous studies have confirmed the activation of the TGF-β1/Smad signaling pathway in DCM [15,16,17]. PANoptosis, a form of innate immune-mediated inflammatory cell death, represents a coordinated process encompassing the features of pyroptosis, apoptosis, and necroptosis [18]. The inhibition of PANoptosis-related sensors, such as NLRP3 and absent in melanoma 2 (AIM2), has been shown to improve cardiac function in DCM rat models [19,20]. Recent studies further implicate the cyclic GMP–AMP synthase (cGAS)–stimulator of interferon genes (STING) signaling pathway in DCM, and significant activation has been observed in mouse models. Notably, STING deficiency improves cardiac dysfunction, likely through the suppression of myocardial inflammation and fibrosis [21,22,23]. In addition, STING knockout has been shown to alleviate lipopolysaccharide-induced lung inflammation and PANoptosis [24]. However, the precise role of PANoptosis in the pathogenesis of DCM remains incompletely understood.

Exercise, as a safe, low-cost, and effective multisystem intervention, has demonstrated substantial benefits in the prevention and treatment of DCM [25]. Swimming exercise has been shown to attenuate myocardial fibrosis in DCM rats [26], and resistance training has been validated to improve cardiac function in diabetic rats [27]. Furthermore, high-intensity interval training (HIIT) has been reported to ameliorate DCM by inhibiting cardiomyocyte apoptosis [28]. However, systematic comparisons of the therapeutic effects of different exercise modalities on DCM remain limited. Moreover, whether exercise exerts its protective effects against DCM through modulation of the cGAS–STING signaling pathway has not been elucidated. Therefore, the present study aimed to investigate the effects of distinct exercise modalities on DCM in mice and to elucidate the underlying mechanisms by which exercise improves cardiac function, reduces inflammation, and suppresses myocardial fibrosis. These findings may provide a scientific basis for identifying the most effective exercise intervention strategy for DCM.

## 2. Methods

### 2.1. Animal Experiments and Study Design

Four-week-old male C57BL/6J mice were obtained from the Model Animal Research Center of Nanjing University (Nanjing, China) and housed in the SPF-grade Animal Experimentation Center of Shanghai University of Sport. The animals were maintained under controlled conditions at 21 ± 2 °C, with relative humidity of 45–50% and a 12 h light phase (8:30 AM–8:30 PM). Following a one-week acclimatization period, the mice were subjected to dietary, intraperitoneal injection, and exercise interventions. All experimental procedures were approved by the ethics committee for Scientific Research at Shanghai University of Sport (Approval No. 102772019DW009) and conducted in compliance with the ARRIVE guidelines.

**Figure 1:**
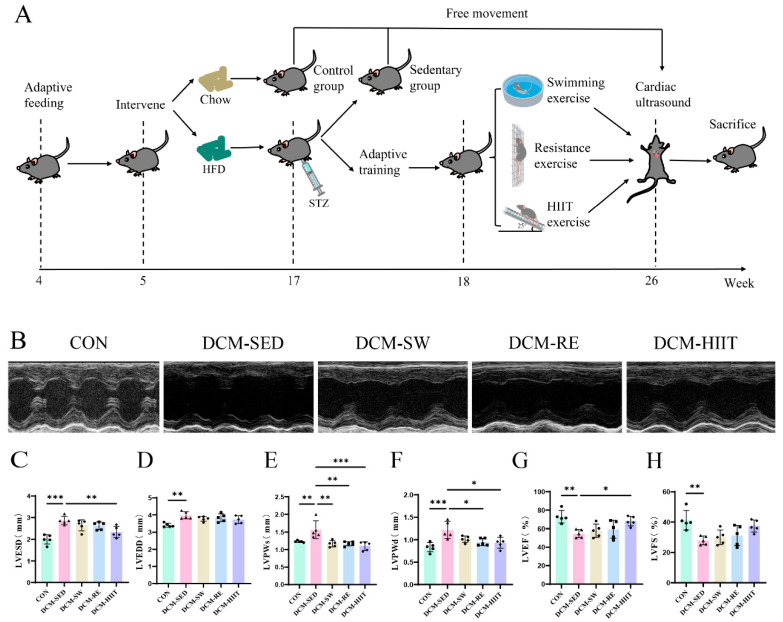
Effects of exercise training on cardiac function in mice: (**A**) Experimental design diagram. (**B**) Representative echocardiographic images. (**C**) LVESD. (**D**) LVEDD. (**E**) LVPWs. (**F**) LVPWd. (**G**) LVEF. (**H**) LVFS, *n* = 5. * *p <* 0.05, ** *p <* 0.01, *** *p <* 0.001.

As described in our previous studies [29,30,31], mice in the control (CON) group were fed a standard chow diet (D12450J, 3.85 kcal/g, 10% fat, 20% protein; SYSE Ltd., Nanjing, China), whereas those in the HFD group received a HFD (D12492, 5.24 kcal/g, 60% fat, 20% protein; SYSE Ltd., Nanjing, China). After 12 weeks of feeding, STZ (S0130, Sigma-Aldrich, St. Louis, MO, USA) was dissolved in 0.1 mmol/L sodium citrate buffer, and mice in the HFD group were intraperitoneally injected with a single dose of STZ (100 mg/kg). Mice in the CON group received an equal volume of sodium citrate buffer. On the seventh day following STZ injection, fasting blood glucose, glucose tolerance, and insulin tolerance were assessed. Mice with fasting blood glucose levels ≥16.7 mmol/L were considered successfully induced with T2DM and were subsequently randomized into one of four groups: sedentary DCM (DCM-SED), swimming exercise (DCM-SW), resistance exercise (DCM-RE), or HIIT (DCM-HIIT). Each group consisted of nine mice, housed four to five per cage. The experimental design is shown in Figure 1A.

### 2.2. Exercise Training Protocol

The exercise training protocols were adapted from our previously published research [30]. Mice in the three exercise groups first underwent a one-week period of adaptive training, followed by eight weeks of formal exercise intervention. Throughout the intervention period, all mice continued to receive HFD feeding (Figure 1A).

Mice in the DCM-SW group underwent non-weight-bearing swimming training in a plastic tank (60 cm × 50 cm × 50 cm) filled with water to a depth of 45 cm, maintained at approximately 35 °C. During the first week, swimming duration was progressively increased from 10 min to 60 min per day, with an increment of 10 min per day. From the second week onward, mice swam for 60 min per day, five days per week, for a total duration of eight weeks.

Mice in the DCM-RE group performed weighted ladder climbing training on a 1 m ladder with 2 cm rung intervals and an inclination angle of 85°. On the first day of the adaptation period, mice climbed without an additional load to acclimate to the apparatus. The load was progressively increased during the adaptation week, reaching 30% of body weight by day 5. From the second week onward, formal training was initiated with an initial load of 30% of body weight. Each session consisted of five climbs with a one-minute rest between climbs, repeated four times with a two-minute rest between sets, lasting approximately 60 min per day. Training was conducted five days per week for eight weeks, during which the load was gradually increased until it reached 100% of body weight.

Mice in the DCM-HIIT group underwent treadmill training at a 25° incline. During the adaptation period, both treadmill speed and incline were gradually increased. In the formal training phase, each session began with a 10 min warm-up at 5 m/min, followed by 10 bouts of high-intensity interval running lasting 4 min each, with 2 min rest intervals after every two bouts. The running speed was progressively increased from 16 m per minute to 26 m per minute over the course of the training period. Training was conducted five days per week for a total of eight weeks.

### 2.3. Echocardiographic Measurements

Following the final training session, a glucose tolerance test (GTT) was performed. After 48 h, echocardiographic assessment was conducted. Mice were anesthetized with 2% isoflurane mixed with 1 L/min O_2_ and placed on a thermostatically controlled heating pad maintained at 37 °C. Cardiac function was evaluated using a small-animal ultrasound system (Vevo1100, Visual Sonics, Toronto, ON, Canada) in both B- and M-modes. The short-axis images of the heart were acquired and stored for subsequent measurements, including left ventricular end-diastolic diameter (LVEDD), left ventricular end-systolic diameter (LVESD), left ventricular posterior wall thickness at end-systole (LVPWs), left ventricular posterior wall thickness at end-diastole (LVPWd), left ventricular ejection fraction (LVEF), and left ventricular fractional shortening (LVFS).

### 2.4. Tissue Collection

On the day following echocardiographic assessment, body weight was recorded prior to tissue collection. After euthanasia, hearts were rapidly excised using sterilized scissors and forceps. The left and right atrial appendages and the right ventricle were removed, and the weights of the whole heart, left ventricle, and right ventricle were measured. The upper one-third of the left ventricle was fixed in 4% paraformaldehyde in a 15 mL centrifuge tube for subsequent morphological analysis, while the remaining portion was placed in 2 mL EP tubes, snap-frozen in liquid nitrogen, and stored at −80 °C for later analyses. Tibia length was measured for each mouse.

### 2.5. Histology

After fixation in 4% paraformaldehyde for 48 h, heart tissues were rinsed with phosphate-buffered saline (PBS) and running water, followed by graded ethanol dehydration, xylene clearing, paraffin infiltration, and embedding. Paraffin blocks were sectioned to prepare tissue sections, which were stained with hematoxylin–eosin (HE) for morphological evaluation and Sirius Red for collagen fiber assessment. Images were acquired under multiple fields using an Olympus microscope, and Sirius Red-positive areas were quantified using ImageJ software (version 1.53, National Institutes of Health, Bethesda, MD, USA).

### 2.6. WGA Staining

Paraffin-embedded heart sections were deparaffinized, rehydrated, washed, and subjected to antigen retrieval. Wheat germ agglutinin (WGA) staining was then performed in the dark, followed by mounting with a DAPI-containing medium. Myocardial sections were examined using an LSM700 laser confocal microscope, and six random fields per section were imaged and recorded. The cross-sectional area of cardiomyocytes was quantified using ImageJ software, and at least 40 cardiomyocytes were measured per field for analysis.

### 2.7. TUNEL Staining

Paraffin-embedded mouse heart sections were deparaffinized with xylene, rehydrated through a graded ethanol series, and treated with proteinase K according to the manufacturer’s instructions. Cardiomyocyte apoptosis was detected using a terminal deoxynucleotidyl transferase-mediated dUTP nick-end labeling (TUNEL) assay kit (C1086, Beyotime, Shanghai, China), and staining results were examined with an LSM700 laser confocal microscope.

### 2.8. Immunofluorescence Staining

After deparaffinization, rehydration through a graded ethanol series, washing with deionized water, and antigen retrieval, paraffin-embedded heart sections were blocked at room temperature for 90 min. Sections were then incubated overnight at 4 °C with primary antibodies against fibronectin (1:300, Santa Cruz Biotechnology, Dallas, TX, USA, sc-8422), Col3a1 (1:300, Santa Cruz Biotechnology, Dallas, TX, USA, sc-271249), α-SMA (1:300, Santa Cruz Biotechnology, Dallas, TX, USA, sc-53142), and STING (1:300, Proteintech, Wuhan, China, 19851-1-AP). On the following day, after removal of the primary antibodies, sections were incubated with fluorescent secondary antibodies (1:300, diluted in 1% BSA-TBST) for 60 min at room temperature in the dark. Nuclei were counterstained with DAPI using an anti-fade mounting medium, and images were acquired with an LSM700 laser confocal microscope. Representative micrographs are presented.

### 2.9. Quantitative Real-Time PCR

Approximately 20 mg of heart tissue was weighed, and total RNA was extracted using the Trizol method. Among them, 1 μg of total RNA was used for cDNA synthesis. And then, cDNA was synthesized using a cDNA Synthesis Kit (AG11706, ACCURATE BIOTECHNOLOGY (HUNAN) Co., Ltd., Changsha, China) following the manufacturer’s protocol. Quantitative real-time PCR (qRT-PCR) was performed using the SYBR Green Premix Pro Tag HS qPCR Kit (Rox Plus) (AG11701, ACCURATE BIOTECHNOLOGY (HUNAN) Co., Ltd,, Changsha, China). The relative mRNA expression levels of target genes were calculated using the 2^−ΔΔCt^ method, with β-actin serving as the internal reference gene. Primer sequences are listed in Table 1.

### 2.10. Western Blotting

Approximately 20 mg of heart tissue was collected and placed in a 2 mL EP tube, followed by the addition of 500 μL of protein lysis buffer (Beyotime, Shanghai, China, P0013B). After homogenization, lysis, and centrifugation, the supernatant was collected, and the protein concentration was determined using a BCA protein assay kit (Beyotime, Shanghai, China, P0010). A total of 30–40 μg of protein from each sample was loaded per well for SDS-PAGE electrophoresis, and the separated proteins were transferred onto PVDF membranes. The membranes were blocked with 5% non-fat milk in TBST for 90 min at room temperature. Subsequently, the membranes were incubated overnight at 4 °C on a shaker with the following primary antibodies: TGF-β1 (1:1000, Santa Cruz Biotechnology, Dallas, TX, USA, sc-52893), α-SMA (1:1000, Santa Cruz Biotechnology, Dallas, TX, USA, sc-53142), ZBP1 (1:1000, Santa Cruz Biotechnology, Dallas, TX, USA, sc-271483), Cleaved-GSDMD/GSDMD (1:1000, Abcam, Cambridge, UK, ab209845), Cleaved-caspase3 (1:1000, Cell Signaling Technology, Boston, MA, USA, 9661), p-MLKL (1:1000, Cell Signaling Technology, Boston, MA, USA, 37333T), MLKL (1:1000, Cell Signaling Technology, Boston, MA, USA, 37705S), cGAS (1:1000, Cell Signaling Technology, Boston, MA, USA,31659S), STING (1:1000, Proteintech, Wuhan, China, 19851-1-AP), TBK1 (1:1000, Abcam, Cambridge, UK, ab40676), and β-actin (1:20000, Proteintech, Wuhan, China, 66009-1-Ig). On the following day, the membranes were incubated with HRP-conjugated secondary antibodies at room temperature for 90 min. Protein bands were visualized using a chemiluminescence imaging system, and the expression levels of target proteins were quantified by calculating the ratio of the target band intensity to that of the internal control.

### 2.11. Statistical Analysis

Statistical analyses were performed using IBM SPSS 27.0 software (International Business Machines Corporation, Armonk, NY, USA). Data are presented as mean ± standard deviation. Homogeneity of variances was tested prior to analysis. One-way analysis of variance was used for comparisons among multiple groups. Sirius Red staining, Western blot, and immunofluorescence images were analyzed using ImageJ software, and graphs were generated with GraphPad Prism 10. A *p*-value < 0.05 was considered statistically significant, while *p* < 0.01 was considered highly significant.

## 3. Results

### 3.1. Exercise Training Improves Cardiac Dysfunction in Mice with DCM

Numerous studies have demonstrated that DCM is associated with marked cardiac dysfunction [32,33,34]. Therefore, we evaluated cardiac function in mice using echocardiography. Compared with the CON group, the DCM group exhibited considerably increased LVESD, LVEDD, LVPWs, and LVPWd (Figure 1B–F). All three exercise interventions effectively attenuated these pathological increases in cardiac parameters (Figure 1B–F). Furthermore, LVEF and LVFS were considerably reduced in the DCM group relative to the CON group, whereas exercise interventions markedly improved both indices (Figure 1G,H). Among the exercise modalities, HIIT produced the most pronounced improvement in cardiac function (Figure 1G,H).

### 3.2. Exercise Training Suppresses Myocardial Inflammation in DCM Mice

**Figure 2:**
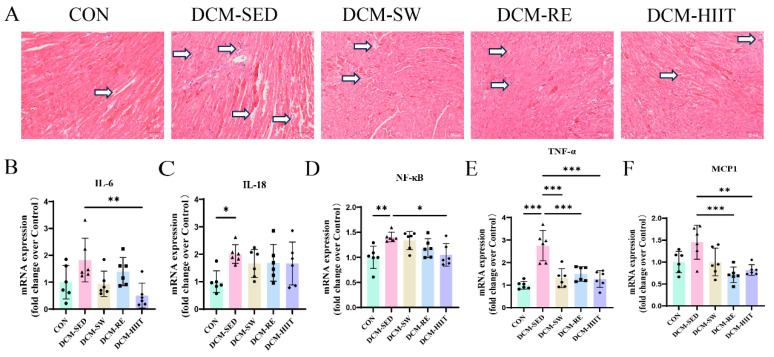
Effects of exercise training on myocardial inflammation in mice: (**A**) Representative images of HE staining of myocardial tissue. Scale bars = 50 μm. (**B**–**F**) mRNA expression levels of inflammatory markers IL-6, IL-18, NF-κB, TNF-α, and MCP1 in myocardial tissue, *n* = 6. * *p <* 0.05, ** *p <* 0.01, *** *p <* 0.001.

To evaluate the effects of exercise training on myocardial structure in DCM mice, HE staining was performed on cardiac tissue. Myocardial cells in the DCM group exhibited disorganized architecture, fractured muscle fibers, and inflammatory cell infiltration, whereas all three exercise modalities alleviated these structural abnormalities (Figure 2A). Analysis of inflammatory mediators showed that IL-6 and MCP1 expression did not differ considerably between the CON and DCM groups (Figure 2B,F). However, compared with the DCM group, all exercise interventions considerably reduced IL-6 and MCP1 expression (Figure 2B,F). Furthermore, pro-inflammatory markers including IL-18, NF-κB, and TNF-α were markedly upregulated in the myocardium of DCM mice relative to the CON group, while each exercise modality considerably suppressed their expression (Figure 2C–E). Among the interventions, HIIT exerted the strongest anti-inflammatory effects (Figure 2C–E). Collectively, these findings indicate that eight weeks of exercise training ameliorates myocardial structural injury and attenuates inflammation in HFD/STZ-induced DCM mice.

### 3.3. Exercise Training Attenuates Myocardial Hypertrophy in DCM Mice

**Figure 3:**
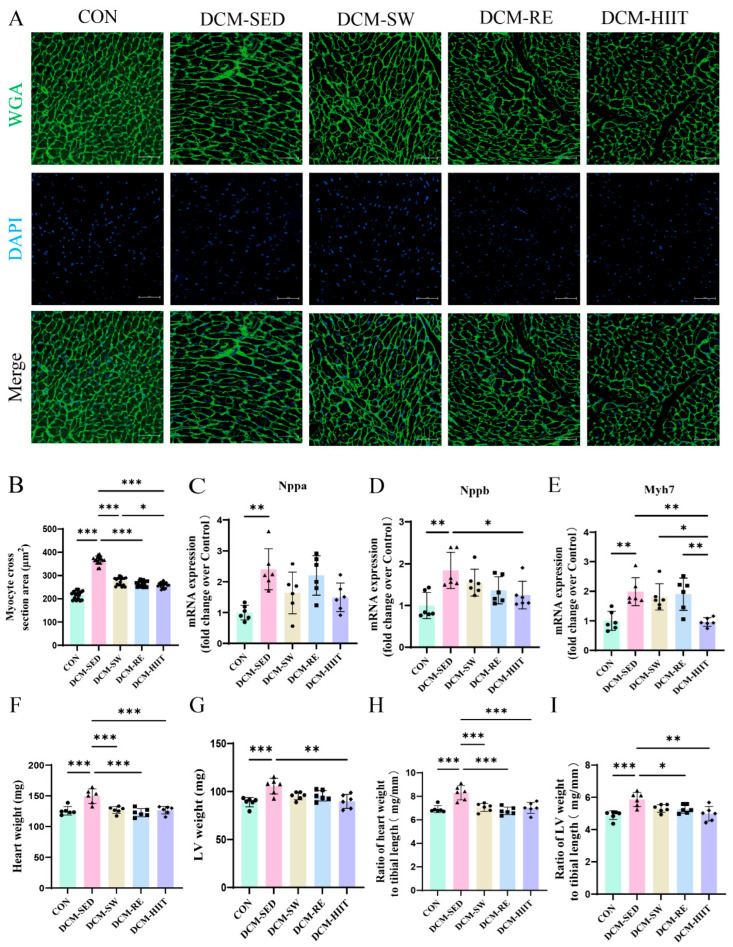
Effects of exercise training on myocardial hypertrophy in mice: (**A**) WGA staining of cardiac tissue. Scale bars = 50 μm. (**B**) Quantification of WGA-stained cardiomyocyte cross-sectional area. Three samples per group were analyzed; for each sample, six fields were randomly selected, and at least 40 cells per field were measured. (**C**–**E**) mRNA expression levels of hypertrophy-related genes Nppa, Nppb, and Myh7 in cardiac tissue. (**F**) Heart weight (HW). (**G**) Left ventricular weight (LVW). (**H**) Ratio of heart weight to tibial length (HW/TL). (**I**) Ratio of Left ventricular weight to tibial length (LVW/TL), *n* = 6. * *p <* 0.05, ** *p <* 0.01, *** *p <* 0.001.

WGA staining was performed to evaluate myocardial hypertrophy in mice. Compared with the CON group, the DCM group exhibited a considerable increase in cardiomyocyte cross-sectional area, whereas all three exercise interventions effectively reduced this hypertrophic change (Figure 3A,B). qRT-PCR analysis further demonstrated that the expression of hypertrophy-related markers, including natriuretic peptide A (Nppa), Nppb, and myosin heavy chain 7 (Myh7), was markedly elevated in the DCM group relative to the CON group, while all exercise modalities considerably suppressed their expression (Figure 3C–E). Consistent with these findings, heart weight (HW), left ventricular weight (LVW), HW/tibial length (HW/TL), and LVW/TL were considerably increased in DCM mice compared with controls, and these indices were reduced by all exercise interventions (Figure 3F–I). Among the modalities, HIIT produced the most pronounced improvement. Collectively, these results indicate that exercise training attenuated myocardial hypertrophy in DCM mice and HIIT provided the greatest therapeutic benefit.

### 3.4. Exercise Training Ameliorates Myocardial Fibrosis in DCM Mice

**Figure 4:**
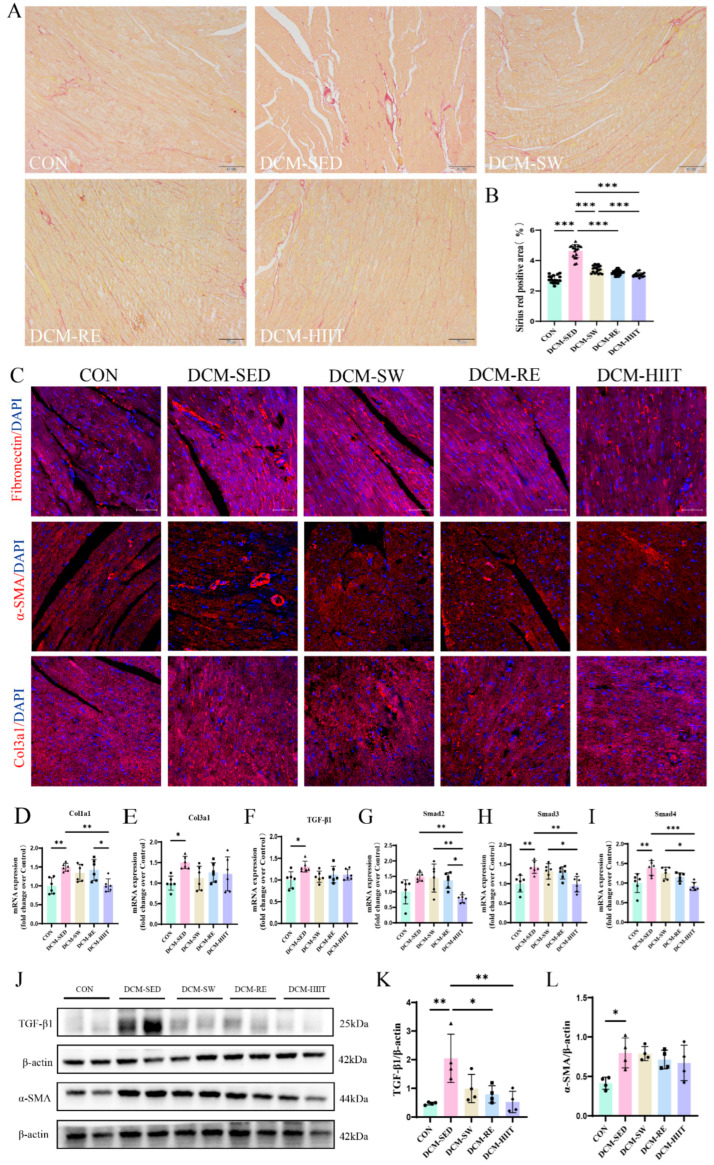
The effects of exercise training on myocardial fibrosis in mice: (**A**) Representative images of Sirius Red staining. Scale bars = 50 μm. (**B**) Quantification of SR staining. For each group, three samples were analyzed, and six randomly selected fields from each sample were used for quantification. (**C**) Representative immunofluorescence images of myocardial fibronectin, α-SMA, and Col3a1. Scale bars = 50 μm. (**D**–**I**) mRNA expression levels of fibrosis-related genes Col1a1, Col3a1, TGF-β1, Smad2, Smad3, and Smad4 in myocardial tissue, *n* = 6. (**J**) Protein expression of TGF-β1, α-SMA, and reference protein β-actin in myocardial tissue. (**K**) Quantitative analysis of TGF-β1 protein expression, *n* = 4. (**L**) Quantitative analysis of α-SMA protein expression, *n* = 4. * *p <* 0.05, ** *p <* 0.01, *** *p <* 0.001. Original images of (**J**) can be found in Supplementary Materials.

Previous studies have demonstrated that DCM is associated with myocardial fibrosis [35]. Consistent with this, Sirius Red staining revealed a considerable increase in collagen fiber deposition in the myocardium of DCM mice compared with the CON group, which was attenuated by all three exercise modalities (Figure 4A,B). Immunofluorescence staining further showed increased positive areas for fibronectin, α-SMA, and Col3a1 in the myocardium of DCM mice, whereas exercise interventions markedly reduced the expression of these fibrosis-related proteins (Figure 4C). qRT-PCR analysis demonstrated that the mRNA expression levels of Col1a1, Col3a1, TGF-β1, Smad2, Smad3, and Smad4 were considerably upregulated in DCM mice relative to controls, whereas all three exercise modalities downregulated their expression, and HIIT exerted the most pronounced inhibitory effect (Figure 4D–I). Consistently, Western blot analysis showed elevated protein levels of TGF-β1 and α-SMA in DCM mice compared with controls, both of which were considerably reduced by exercise training, and no statistical differences were observed among exercise types (Figure 4J–L). Collectively, these results indicate that exercise training mitigates myocardial fibrosis in DCM mice by suppressing extracellular matrix deposition, and HIIT demonstrated the greatest efficacy.

### 3.5. Exercise Training Suppresses PANoptosis in the Myocardium of DCM Mice

**Figure 5:**
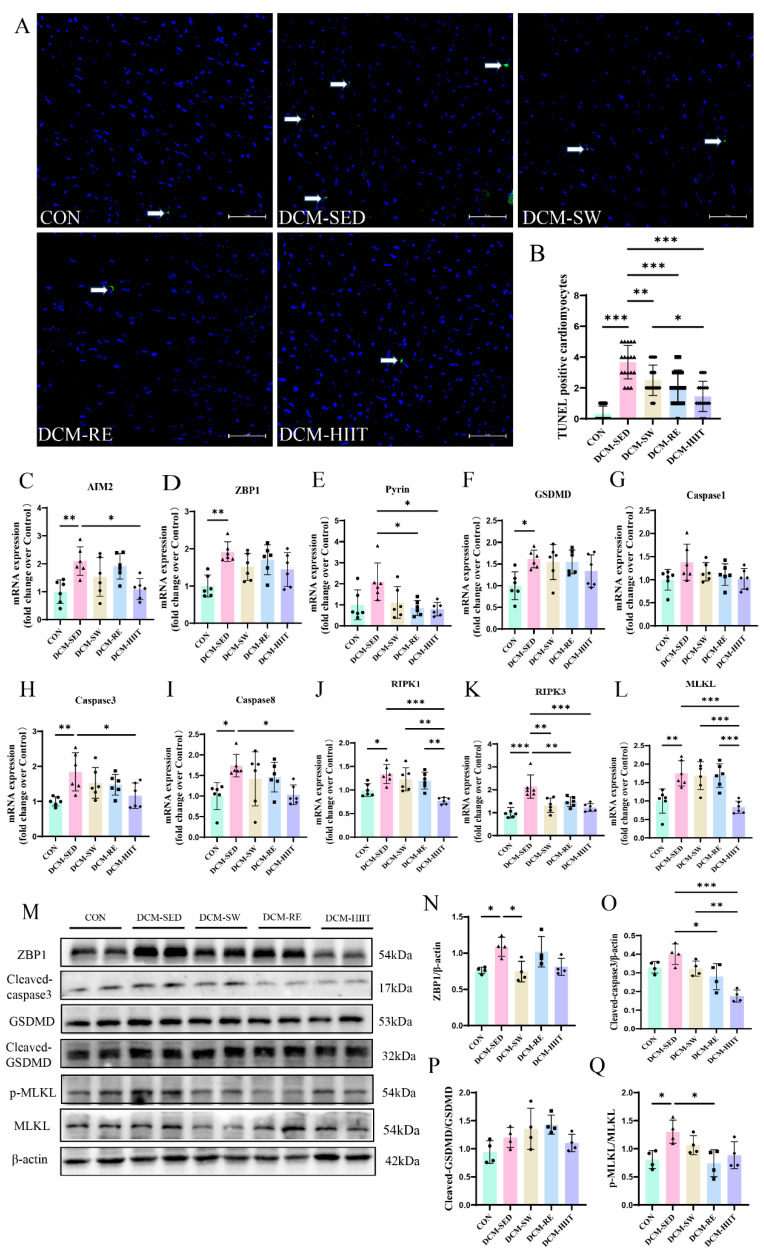
Effects of exercise training on cardiomyocyte PANoptosis in mice: (**A**) Representative TUNEL staining images of cardiac tissue. Scale bars = 50 μm. (**B**) Quantification of TUNEL-positive cells. For each group, three samples were analyzed, and six randomly selected fields from each sample were used for quantification. (**C**–**L**) mRNA expression levels of PANoptosis-related genes including AIM2, ZBP1, Pyrin, GSDMD, Caspase1, Caspase3, Caspase8, RIPK1, RIPK3, and MLKL in cardiac tissue, *n* = 6. (**M**) Representative Western blot images showing protein expression of ZBP1, Cleaved-caspase3, GSDMD, Cleaved-GSDMD, p-MLKL, MLKL, and the loading control β-actin in cardiac tissue. (**N**) Quantification of ZBP1 protein expression, *n* = 4. (**O**) Quantification of Cleaved-caspase3 protein expression, *n* = 4. (**P**) Quantification of the Cleaved-GSDMD/GSDMD protein ratio, *n* = 4. (**Q**) Quantification of the p-MLKL/MLKL protein ratio, *n* = 4. * *p <* 0.05, ** *p <* 0.01, *** *p <* 0.001. Original images of (**M**) can be found in Supplementary Materials.

Previous studies have implicated PANoptosis in the pathogenesis of DCM [36,37]. In line with these findings, our results showed that the number of TUNEL-positive apoptotic cardiomyocytes was markedly increased in the DCM group compared with the CON group (Figure 5A,B). All three types of exercise interventions considerably reduced cardiomyocyte apoptosis in DCM mice (Figure 5A,B). Furthermore, the mRNA expression levels of PANoptosis-related markers, including AIM2, Z-DNA binding protein 1 (ZBP1), pyrin, caspase-1, gasdermin D (GSDMD), caspase-3, caspase-8, mixed lineage kinase domain-like pseudokinase (MLKL), receptor-interacting protein kinase 1 (RIPK1), and RIPK3, were considerably elevated in the myocardium of DCM mice compared with controls, while exercise training effectively suppressed their expression (Figure 5C–L). Consistently, protein levels of ZBP1, cleaved-caspase-3, cleaved-GSDMD/GSDMD, and p-MLKL/MLKL were elevated in DCM mice but decreased following exercise interventions (Figure 5M–O,Q). Notably, only HIIT considerably reduced the Cleaved-GSDMD/GSDMD ratio in DCM mice (Figure 5M,P). Together, these findings suggest that exercise training attenuates PANoptosis in the myocardium of DCM mice, and HIIT exerted the strongest inhibitory effect.

### 3.6. Exercise Training Inhibits Activation of the cGAS-STING Pathway in the Myocardium of DCM Mice

**Figure 6:**
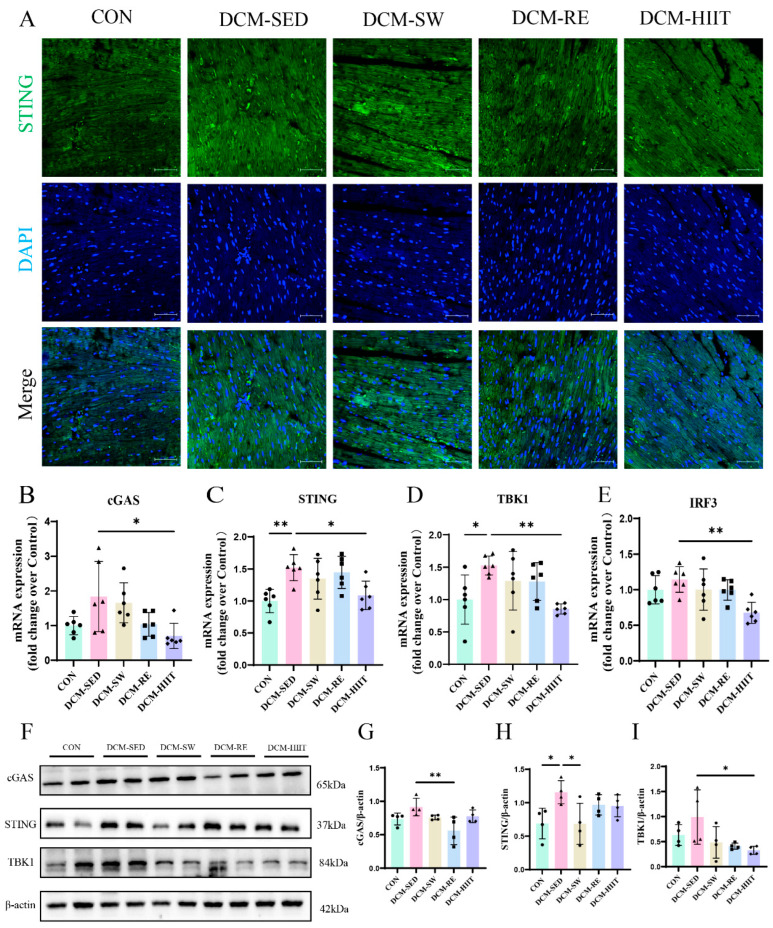
Effects of exercise training on the myocardial cGAS-STING pathway in mice: (**A**) Representative immunofluorescence images of STING in myocardial tissue. Scale bars = 50 μm. (**B**–**E**) mRNA expression levels of cGAS, STING, TBK1, and IRF3 in myocardial tissue, *n* = 6. (**F**) Protein expression levels of cGAS, STING, TBK1, and the internal control β-actin in myocardial tissue. (**G**) Quantitative analysis of cGAS protein levels, *n* = 4. (**H**) Quantitative analysis of STING protein levels, *n* = 4. (**I**) Quantitative analysis of TBK1 protein levels, *n* = 4. * *p <* 0.05, ** *p <* 0.01. Original images of (**F**) can be found in Supplementary Materials.

To further explore the molecular mechanisms underlying the beneficial effects of exercise training on cardiac function in DCM mice, we assessed the expression of key components of the cGAS–STING pathway at both the mRNA and protein levels. Immunofluorescence staining demonstrated a marked increase in STING-positive areas in the myocardium of DCM mice compared with the CON group, whereas all three exercise interventions markedly reduced STING-positive staining (Figure 6A). In agreement, qRT-PCR analysis revealed that mRNA levels of cGAS, STING, TANK-binding kinase 1 (TBK1), and interferon regulatory factor 3 (IRF3) were considerably elevated in the DCM group relative to controls, but were effectively suppressed by exercise training, and HIIT exerted the strongest inhibitory effect (Figure 6B–E). Consistently, Western blot analysis showed increased protein expression of cGAS, STING, and TBK1 in DCM mice, which was markedly downregulated following all three exercise regimens (Figure 6F–I). Collectively, these findings suggest that exercise training mitigates cardiac dysfunction in DCM mice, at least in part, by inhibiting activation of the cGAS–STING pathway, and HIIT demonstrated the most pronounced effect.

## 4. Discussion

This study aimed to investigate the effects of different exercise modalities on DCM in mice and to elucidate potential underlying mechanisms. A DCM model was established by feeding mice an HFD combined with a single intraperitoneal injection of STZ. The mice were then randomly allocated into four groups: sedentary control, swimming exercise, resistance exercise, and HIIT. We systematically evaluated the impact of these exercise interventions on cardiac function, myocardial hypertrophy, inflammation, fibrosis, PANoptosis, and activation of the cGAS–STING signaling pathway in DCM mice.

In this study, DCM mice displayed left ventricular dysfunction, characterized by reduced LVEF and LVFS, along with increased LVESD and LVEDD, indicating impaired systolic and diastolic function. Myocardial hypertrophy was evident, as shown by enlarged cardiomyocyte cross-sectional area, elevated LVW and LVW/TL, and upregulation of hypertrophy-related genes, including Nppa, Nppb, and Myh7. All three exercise modalities effectively reversed these pathological changes, and HIIT exerted the most pronounced effects in restoring cardiac function and reducing myocardial hypertrophy. Previous studies have consistently reported that patients with DCM exhibit cardiac dysfunction [3,38,39], while in vivo models similarly demonstrate increased LVEDD and LVPWd with reduced LVEF and LVFS [33,40]. Exercise training has been shown to improve cardiac dysfunction in DCM rats through modulation of myocardial metabolites [41]. Moreover, myocardial hypertrophy is a hallmark pathological feature of DCM [42], and earlier studies have demonstrated that HFD-fed C57BL/6J mice exhibit elevated hypertrophy markers such as ANP, BNP, and β-MHC, while 8 weeks of HIIT alleviates myocardial hypertrophy in aged DCM rats [43,44]. Our findings are consistent with these reports and further highlight the therapeutic potential of exercise in mitigating cardiac dysfunction and hypertrophy in DCM. Notably, the superior efficacy of HIIT may be attributed to enhanced myocardial adaptive responses induced by its higher training intensity, providing valuable insights for tailoring individualized exercise interventions in DCM.

Our findings revealed that myocardial fibers in DCM mice were markedly disorganized, with evidence of rupture and inflammatory cell infiltration. These pathological changes were attenuated by all three exercise interventions. Consistently, DCM mice exhibited considerably elevated myocardial expression of inflammatory mediators, including IL-6, IL-18, TNF-α, NF-κB, and MCP1, whereas exercise training suppressed their expression, and HIIT demonstrated the most pronounced inhibitory effects. Inflammation is widely recognized as a key driver in the progression of DCM. Pro-inflammatory cytokines released by immune cells, such as TNF-α and IL-6, exert direct effects on cardiomyocytes and resident cardiac fibroblasts, inducing apoptosis and dysfunction, impairing endothelial barrier integrity, promoting fibroblast activation, and facilitating collagen deposition. Collectively, these processes exacerbate myocardial dysfunction and contribute to the onset and progression of DCM [45,46,47]. In line with previous work by Shabab et al. [48], who showed that exercise training attenuates cardiac injury in DCM rats through suppression of inflammation, our results confirm that exercise alleviates myocardial inflammation in DCM mice. Moreover, our data highlight the relative advantage of HIIT, which exerted the strongest anti-inflammatory effects among the tested modalities.

Our results demonstrated significant collagen deposition in the myocardium of DCM mice, accompanied by elevated expression of Col1a1, Col3a1, fibronectin, and α-SMA, along with the robust activation of the TGF-β1/Smad signaling pathway. All three exercise interventions attenuated collagen accumulation and downregulated fibrosis-related markers, and HIIT exerted the strongest inhibitory effects. Under conditions of chronic inflammation, immune cells release pro-inflammatory cytokines that activate fibroblasts, which subsequently differentiate into myofibroblasts. These myofibroblasts display enhanced proliferative and secretory capacity, thereby driving extracellular matrix remodeling and excessive collagen deposition, ultimately leading to myocardial fibrosis [49,50]. Fibrosis is a hallmark pathological feature of DCM that impairs both systolic and diastolic function, contributing to the progression toward heart failure [51]. Among the signaling pathways involved, TGF-β is a central mediator of fibrosis, promoting myocardial remodeling through both Smad-dependent and -independent pathways [52,53]. Previous studies have similarly reported increased myocardial expression of Col1a1 and Col3a1 in DCM mice [54]. Notably, moderate-intensity aerobic exercise has been shown to attenuate myocardial fibrosis in diabetic mice [55], and some studies suggest that moderate-intensity continuous training surpasses HIIT in reducing fibrosis in DCM rats [56]. By contrast, our findings indicate that swimming, resistance training, and HIIT all alleviated myocardial fibrosis in DCM mice, and HIIT conferred the most pronounced antifibrotic effect.

As an integrated form of programmed cell death that encompasses pyroptosis, apoptosis, and necroptosis, PANoptosis has recently emerged as a critical mechanism in cardiovascular diseases [18]. To the best of our knowledge, this is the first study to systematically assess PANoptosis in the myocardium of DCM mice and to evaluate the modulatory effects of exercise training. We observed a marked increase in TUNEL-positive cardiomyocytes in DCM mice, accompanied by upregulation of PANoptosis-associated sensors (AIM2, ZBP1, and pyrin), apoptotic markers (caspase-3 and caspase-8), pyroptotic mediators (caspase-1 and GSDMD), and necroptotic effectors (RIPK1, RIPK3, and MLKL). These findings indicate that PANoptosis is actively engaged in the pathogenesis of DCM. Importantly, all three exercise modalities significantly downregulated the expression of these markers, and HIIT exerted the most potent inhibitory effect. Previous work has suggested that PANoptosis may exacerbate diabetes by promoting inflammation through the release of intracellular danger signals such as HMGB1 and ATP [57]. In line with this, genetic ablation of ADAM17—a regulator of PANoptosis—has been reported to attenuate cardiomyocyte apoptosis, improve ventricular remodeling, and preserve cardiac function in DCM mice, potentially via suppression of ACE2, activation of the AMPK pathway, and TFEB-mediated enhancement of autophagy [36,37]. These findings suggest a potential role of PANoptosis in the pathogenesis and progression of DCM. Exercise training, as a safe and nonpharmacological intervention, has been implicated in the regulation of PANoptosis. For example, it has been shown to increase skeletal muscle secretion of L-β-aminoisobutyric acid, which can suppress PANoptosis in nucleus pulposus cells through activation of the AMPK/NF-κB pathway [58]. Extending these observations, our study demonstrates for the first time that exercise training, particularly HIIT, effectively inhibits myocardial PANoptosis in DCM mice.

Pathogenic factors such as viral infection can induce DNA damage and trigger the cGAS–STING signaling pathway, leading to enhanced production of IFN-β and nuclear translocation of NF-κB, thereby initiating inflammatory responses [59,60]. In the present study, we observed a significant upregulation of cGAS, STING, TBK1, and IRF3 expression in the myocardia of DCM mice, suggesting that aberrant activation of this pathway is involved in DCM progression. All three types of exercise training suppressed cGAS–STING activation, and HIIT exerted the most pronounced effect. Our findings are consistent with previous reports demonstrating abnormal activation of the cGAS–STING pathway in DCM mice [61,62]. Notably, cGAS–STING signaling has been closely linked to cardiac inflammation, fibrosis, and cardiomyocyte death. For example, free fatty acid-induced mitochondrial damage and the consequent release of mtDNA can activate cGAS–STING, thereby promoting pyroptosis and pro-inflammatory responses through an NLRP3 inflammasome-dependent mechanism and ultimately contributing to myocardial hypertrophy in DCM [22]. In vitro, high-glucose stimulation has been shown to increase STING expression in neonatal ventricular cardiomyocytes, concomitant with α-SMA upregulation, whereas pharmacological inhibition of STING with C-176 partially attenuated cardiomyocyte fibrosis [63]. Moreover, Wang et al. reported that cGAS–STING signaling can promote PANoptosis in HT22 cells via regulation of endoplasmic reticulum stress [64], highlighting its role as an upstream regulator of multiple pathogenic processes. Exercise training has emerged as an effective nonpharmacological strategy to suppress cGAS–STING signaling and mitigate metabolic disorder-associated organ injury. Aerobic exercise has been shown to alleviate metabolic dysfunction-associated fatty liver disease by upregulating the adipokine Nrg4 and inhibiting hepatic cGAS–STING activation [65]. Similarly, aerobic training attenuated HFD-induced cardiac dysfunction, pyroptosis, and inflammation in obese mice through inhibition of the STING–NLRP3 signaling axis [66]. Our study is the first to demonstrate that multiple forms of exercise can suppress myocardial cGAS–STING activation in DCM and HIIT showed the most robust effect. These results suggest that modulation of cGAS–STING signaling may represent a novel mechanism underlying the cardioprotective effects of exercise in DCM, and a potential therapeutic target for future intervention.

This study investigated the effects of three different exercise modalities on DCM in mice. However, several limitations should be acknowledged. First, variations in exercise intensity may exist among the three training regimens, which could be more precisely quantified by measuring maximal oxygen uptake or heart rate. Second, while we examined inflammatory responses at the transcriptional level, we did not assess protein expression of inflammatory mediators, which may provide a more comprehensive evaluation of the inflammatory state. Third, although we systematically analyzed cardiac function, hypertrophy, fibrosis, inflammation, PANoptosis, and cGAS–STING signaling, the intrinsic mechanistic relationships among these pathological processes remain to be fully clarified. Our findings suggest that the cGAS–STING pathway may act as an upstream driver that activates downstream processes such as inflammation and PANoptosis, ultimately leading to myocardial fibrosis and impaired cardiac function. To validate this hypothesis, future studies employing STING transgenic or knockout mouse models will be necessary to establish, at the genetic level, the causal role of cGAS–STING signaling in mediating the cardioprotective effects of exercise in DCM.

## 5. Conclusions

This study demonstrated that DCM induced by HFD combined with STZ was characterized by activation of the cGAS–STING signaling pathway, accompanied by increased expression of inflammatory cytokines and PANoptosis-related genes, pronounced myocardial fibrosis, and impaired cardiac function. All three types of exercise interventions effectively suppressed cGAS–STING pathway activity, reduced inflammation and PANoptosis, alleviated myocardial fibrosis, and improved cardiac function. Notably, HIIT exerted the most pronounced overall therapeutic effects.

## Figures and Tables

**Table 1:** Primers used for quantitative real-time PCR analysis.

Gene	Forward Primer	Reverse Primer
IL-6	TAGTCCTTCCTACCCCAATTTCC	TTGGTCCTTAGCCACTCCTTC
IL-18	GACTCTTGCGTCAACTTCAAGG	CAGGCTGTCTTTTGTCAACGA
NF-κB	ATGGCAGACGATGATCCCTAC	CGGAATCGAAATCCCCTCTGTT
TNF-α	CCTGTAGCCCACGTCGTAG	GGGAGTAGACAAGGTACAACCC
MCP1	AGGTCCCTGTCATGCTTCTG	TGGGATCATCTTGCTGGTG
Nppa	TGGGATCATCTTGCTGGTG	GGGGGCATGACCTCATCTT
Nppb	AGTCCTTCGGTCTCAAGGCA	CCGATCCGGTCTATCTTGTGC
Myh7	CCTGCGGAAGTCTGAGAAGG	CTCGGGACACGATCTTGGC
Col1a1	CTGGCGGTTCAGGTCCAAT	TTCCAGGCAATCCACGAGC
Col3a1	CTGTAACATGGAAACTGGGGAAA	CCATAGCTGAACTGAAAACCACC
TGF-β1	CTTCAATACGTCAGACATTCGGG	GTAACGCCAGGAATTGTTGCTA
Smad2	AAGCCATCACCACTCAGAATTG	CACTGATCTACCGTATTTGCTGT
Smad3	CATTCCATTCCCGAGAACACTAA	GCTGTGGTTCATCTGGTGGT
Smad4	ACACCAACAAGTAACGATGCC	GCAAAGGTTTCACTTTCCCCA
cGAS	CAGGAAGGAACCGGACAAGC	CCGACTCCCGTTTCTGCATT
STING	GGTCACCGCTCCAAATATGTAG	CAGTAGTCCAAGTTCGTGCGA
TBK1	TGCTGGGGTTTTGACCAGTT	TCTTATGCGCCGTCATGTGT
IRF3	TGGGTCAAGAGGCTTGTGAT	ATGTCCTCCACCAAGTCCTG
AIM2	GTCCTCAAGCTAAGCCTCAGA	CACCGTGACAACAAGTGGAT
ZBP1	GAAATGCCAAGTGCCCAAGAA	CCCGCCTATGCTCCATGTT
Pyrin	TCATCTGCTAAACACCCTGGA	CCCCGTAGTAGGTTATCAGAAGG
GSDMD	TTAATTGAGGCGGCAGACTT	TGGGCTGGTCCTGTAAAATC
Caspase1	ACAAGGCACGGGACCTATG	TCCCAGTCAGTCCTGGAAATG
Caspase3	TGGAGGCTGACTTCCTGTATGC	ATTCCGTTGCCACCTTCCTGTT
Caspase8	TGCTTGGACTACATCCCACAC	TGCAGTCTAGGAAGTTGACCA
RIPK1	GACAGACCTAGACAGCGGAG	CCAGTAGCTTCACCACTCGAC
RIPK3	CCAGAGAGCCAAGCCAAAGAG	AGCCACGGGGTCAGAAGATG
MLKL	TTAGGCCAGCTCATCTATGAACA	TGCACACGGTTTCCTAGACG
β-actin	CGTGCGTGACATCAAAGAGAA	GCTCGTTGCCAATAGTGATGA

## Data Availability

The data that support the findings of this study are available from the corresponding author upon reasonable request due to privacy or ethical restrictions.

## Supplementary Materials

The following supporting information can be downloaded at: https://www.mdpi.com/article/10.3390/biom15091223/s1, Full Original western blot images are provided in Supplementary figures.
