# Supplementary material for: Beneficial Effects of Different Types of Exercise on Diabetic Cardiomyopathy
Source: Biomolecules. 2025 Aug 25;15(9):1223. doi: 10.3390/biom15091223 (PMC12466945; doi:10.3390/biom15091223)

**Figure 4J:**

western blots of TGF-β1:

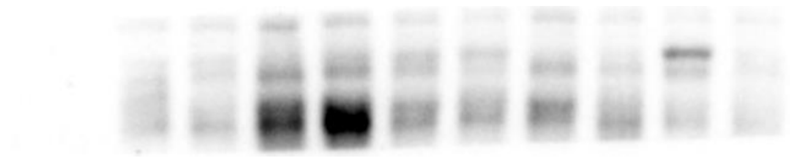

western blots of β-actin:

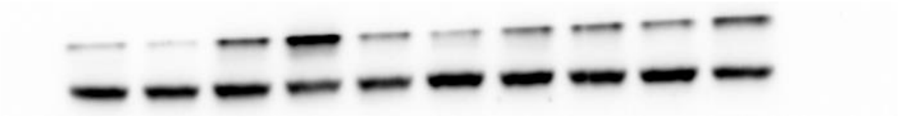

western blots of α-SMA:

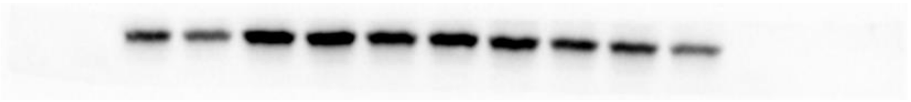

western blots of β-actin:

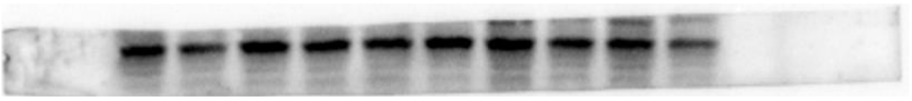

**Figure 5M:**

western blots of ZBP1:

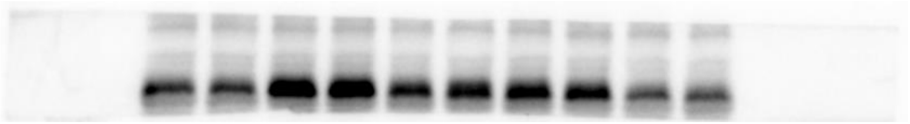

western blots of Cleaved-caspase3:

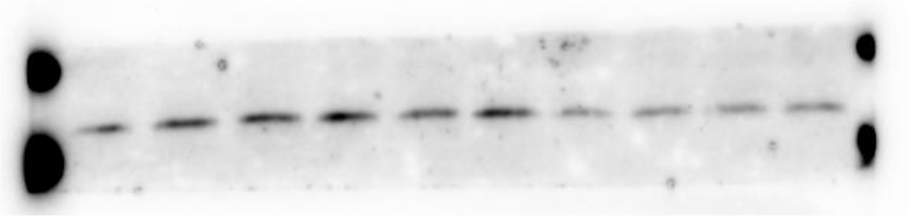

western blots of Cleaved-GSDMD:

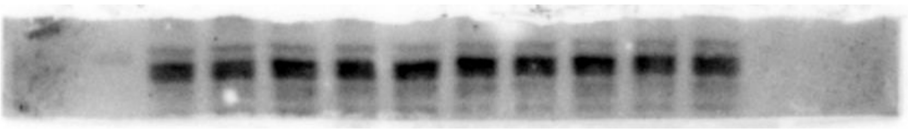

western blots of GSDMD:

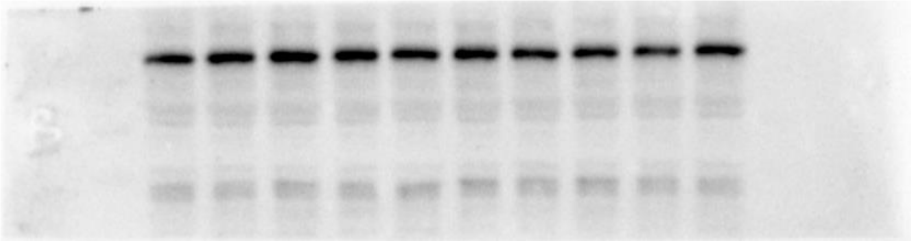

western blots of p-MLKL:

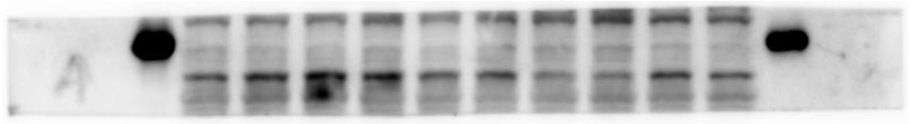

western blots of MLKL:

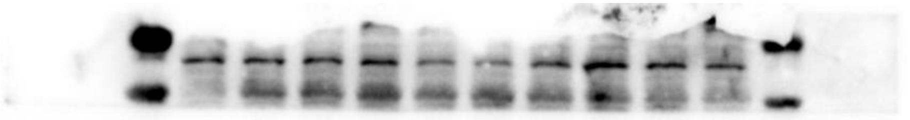

western blots of β-actin:

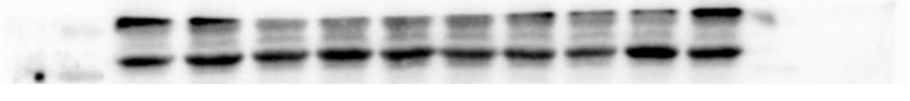

**Figure 6F:**

western blots of cGAS:

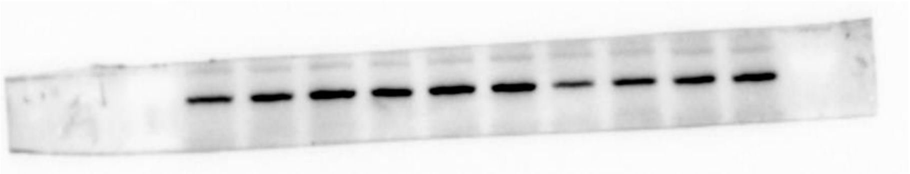

western blots of STING:

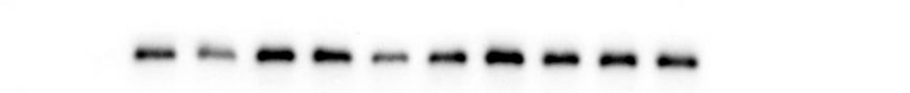

western blots of TBK1:

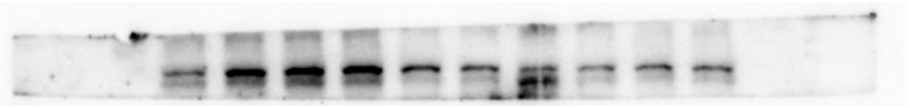

western blots of β-actin:

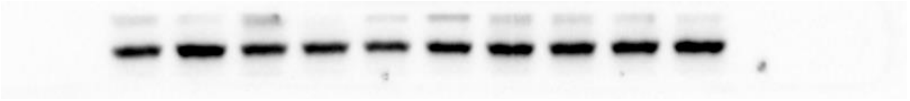

Supplement: Supplementary file 1 [file biomolecules-15-01223-s001.zip › biomolecules-3800332/biomolecules-3800332-supplementary.pdf.pdf]
